# The Antibacterial Properties of Polish Honey against *Streptococcus mutans*—A Causative Agent of Dental Caries

**DOI:** 10.3390/antibiotics12111640

**Published:** 2023-11-19

**Authors:** Dorota Grabek-Lejko, Tomasz Hyrchel

**Affiliations:** Department of Bioenergetics, Food Analysis and Microbiology, Institute of Food Technology and Nutrition, University of Rzeszow, Zelwerowicza 4 Street, 35-601 Rzeszow, Poland; tomek.hyrchel@onet.pl

**Keywords:** dental caries, *S. mutans*, honey, antibacterial, GOX, hydrogen peroxide, antioxidants, polyphenols

## Abstract

*Streptococcus mutans* is considered the main pathogen responsible for dental caries, one of the major infectious diseases, affecting more than 4 billion people worldwide. Honey is a natural product with well-known antibacterial potential against several human pathogens. The aim of the study was to evaluate the antibacterial efficacy of Polish honey against *S. mutans* and analyze the role of some bioactive substances on its antibacterial action. The antibacterial potential of different honey varieties (goldenrod, buckwheat, honeydew, and lime) was analyzed using a microdilution assay. Manuka and artificial honey were used as controls. The content of GOX, hydrogen peroxide, total polyphenols, and antioxidant potential was assayed in honey. The influence of catalase and proteinase K on antibacterial activity as well as antibiofilm action was also determined. The strongest antibacterial activity was observed for buckwheat, honeydew, and manuka honey, which were also characterized by the highest antioxidant activity and polyphenols content. Catalase treatment decreases the antibacterial activity of honey, while proteinase K treatment influences the antibacterial potential of honey slightly less. Obtained results suggest that honey can be a good natural product against *S. mutans,* and hydrogen peroxide was identified as a crucial contributor to its antimicrobial action.

## 1. Introduction

Dental caries (also known as tooth decay or dental cavities) is one of the most common and widespread noncommunicable oral disease, affecting more than 3.5 billion people worldwide [1,2]. A recent epidemiological study showed that the global number of cases of caries of permanent teeth increased by 46.1% from 1990 to 2019 [3]. 

The oral mouth is colonized by 700 to 1000 microbial species, but only some of them are responsible for dental caries [1]. *Streptococcus mutans*, a Gram-positive bacteria, is the main cariogenic pathogen responsible for dental caries when an imbalance in the microbiota occurs. The species’ cariogenic potential is directly related to its metabolic activity and development of mechanisms allowing the bacteria to integrate into the dental biofilm (plaque) and to colonize tooth surfaces. *S. mutans* produces organic acids during diet carbohydrates metabolism, survives low pH conditions, and is able to synthesize extracellular polymeric substances (EPSs). The production of EPSs (like glucans and fructans) from sugars promotes bacterial growth and its adherence to the dental surface, resulting in the formation of a biofilm on tooth surfaces (known as dental plaque) [4]. The cells in biofilm can communicate with each other by secreting specialized proteins and DNA and are more resistant to harsh environments and antibiotics than planktonic cells [4,5,6].

This growing global problem of dental caries needs to be solved. Antibiofilm agents can interrupt at different stages of biofilm production and can inhibit biofilm formation [7,8]. Nowadays, the most important practice to reduce the development of dental caries is to control dental plaque. The prevention of dental plaque can be achieved via mechanical plaque-control tooth brushing and chemical plaque-control mouth washing. Most commercially available mouthwashes utilize fluoride, chlorhexidine, delmopinol, ammonium salts, and essential oils as antimicrobial agents [3,7,9]. However, these substances have some drawbacks: (1) They do not act selectively, affecting both pathogenic species and commensal beneficial species and causing some undesired side effects—vomiting, diarrhea, addiction, teeth discoloration; (2) Long intake and overdose can cause bacteria drug resistance, which is considered a serious health problem nowadays. On the other hand, the removal of bacterial biofilms through brushing needs many repetitions because of the rapid recolonization of the tooth surface [7,10]. Recently, consumers have turned towards the use of natural substances for prophylaxis and the treatment of different diseases, including dental caries; lower prices and fewer side effects are their advantages [11]. Some constituents found in plants like cranberry, *Morus alba*, red wine grapes, barley coffee, curcumin, aloe vera, green tea extract, etc., have been investigated as potential natural agents that can be used against dental caries [12,13]. Among these natural substances, honey can also be considered as a good source of antimicrobial agents, and can be used to fight dental caries. Honey is a naturally sweet substance produced by honey bees from the nectar or secretions of plants or the excretions of plant sucking insects on the living parts of plants, which the bees collect and transform. Honey consists of over 200 different compounds but is mostly composed of sugars (80–85%), water (15–17%), proteins (0.1–0.4%), and other components such as enzymes, organic acids, vitamins, and phenolic compounds. Among honey sugars, the amount of fructose varies from 35.6 to 41.8%, glucose from 25.4 to 28.1%, maltose from 1.8 to 2.7%, and sucrose from 0.23 to 1.21% [14]. Since ancient times, honey has been used not only as a food or sweetener but also in folk medicine for the treatment of many diseases, demonstrating antibacterial, antifungal, antiviral, anticancer, and antidiabetic potential [3,15]. Moreover, several studies have demonstrated its protective effect on cardiovascular, nervous, respiratory, and gastrointestinal systems. Various components contribute to the antimicrobial potential of honey: the high sugar content (osmolarity), low water activity, low pH, peptides, and proteins (defensin-1, glucose oxidase), hydrogen peroxide, phytochemicals–phenolic compounds, and methylglyoxal (MGO) [16,17]. It was demonstrated, in many in vitro and in vivo studies, that honey inhibits the growth of wide spectrum of bacteria, including *Staphylococcus aureus*, *Escherichia coli*, *Pseudomonas aeruginosa*, *Campylobacter jejuni*, *Helicobacter pylori*, *Micrococcus luteus*, and *Bacillus cereus*, as well as multidrug-resistant bacteria like *S. aureus* MRSA [3,15,18,19].

Although the antibacterial potential of honey against different Gram-positive and Gram-negative bacteria has been analyzed and proven by many authors, the antibacterial potential of honey against *S. mutans* (the main causative agent of dental caries) is limited to very few studies, and the results presented therein vary [20,21,22,23,24,25]. Moreover, the high concentration of sugars in honey raises the question of whether honey can actually inhibit the growth of *S. mutans* or, on the contrary, improve the growth of this bacterium and promote formation of dental caries. It is known that the intake of dietary sugars is the most important risk factor for dental caries, but, on the other hand, sugars naturally present in grains, vegetables, fruits, and dairy products do not significantly affect the formation of caries because of other protective factors, like polyphenols, that inhibit its formation [26].

According to our knowledge, there are no data describing the antibacterial potential of honey from Poland against this periodontal pathogen. The aim of the study was to evaluate the antibacterial efficacy of Polish honey collected in the southeastern part of Poland, especially Podkarpacie honeydew honey (which has been on the EU’s protected designation of origin list since 2010), against *S. mutans* and analyze the role of some bioactive substances on its antibacterial action. The possible antibacterial potential was compared with well-known medical-grade manuka honey and artificial honey. 

## 2. Results and Discussion

The variety of nectar honeys, apart from the beekeepers’ declarations (based on floral availability during the harvest season, location of the apiary) was confirmed by pollen analysis. Honeydew honey labeling was confirmed by measuring its electrical conductivity values (the minimum required is 0.8 mS/cm) and sensory judgement, because there is no internationally accepted quality criterion for honeydew honeys [27]. The quality of the tested honeys was confirmed on the basis of physicochemical tests strictly defined in the EU Directive from 2014 [28]. All honey samples met the requirements.

### 2.1. Antioxidant Properties

#### 2.1.1. Total Phenolic Content

Among many substances responsible for the antibacterial potential of honey, phenolic compounds are always present in honey and have been strongly related to its antibacterial properties, promoting its wide application in the prevention and treatment of many diseases [29]. Recent studies have reported that phenolic compounds can generate hydrogen peroxide by reducing metal ions (Fe (III) to Fe (II)) and triggering a Fenton reaction. As a result, reactive oxygen species such as hydroxyl radicals are generated [3,30]. Results of total phenolic content of the analyzed honey are shown in Table 1. The concentration of phenolics are in accordance with our previous results [17] and other authors describing honey collected in Poland [31,32], with the average content of polyphenolic compounds of varietal honeys in the following order: buckwheat > honeydew > lime > goldenrod. Manuka honey, considered as one of the most active honeys, expressed a similar phenolic concentration to the analyzed dark honeys (buckwheat and honeydew). The lowest level of phenolic compounds was observed for artificial honey (Table 1). However, the total phenolics content varied greatly among the honey types; the highest variability was observed for buckwheat and honeydew honey, where the differences between honey samples reached almost 50–60%. Similarly, Puścion-Jakubik et al. [32] reported even higher differences in buckwheat and honeydew honey samples, ranging from 44.95 to 241.87 and 42.8–148.3 mg of gallic acid per 100 g of honey, respectively. The lowest differences were observed for lime and goldenrod honey. Generally, we observed that honeydew and buckwheat honey had two- to threefold higher content of total polyphenols than light honey such as goldenrod and lime. Similar observations were previously described [17,32,33]. In the case of artificial honeys, the content of polyphenolic compounds was comparable to light honeys (lime) or was even higher (compared to goldenrod honey). Similarly, Gośliński et al. [29] observed that the phenolic content of artificial honey is comparable with light honeys, but in some cases is even higher. 

#### 2.1.2. Antioxidant Properties Measured by FRAP Method

Among tested samples, the strongest reducing antioxidant activity measured by FRAP was observed for buckwheat honey, then for honeydew honey (Table 1). The lowest reducing antioxidant potential was observed for lime and goldenrod honey. Manuka honey exhibited a potential similar to dark honeys. However, Gośliński et al. [29] reported that manuka honey showed higher antioxidant potential than buckwheat honey and was comparable to honeydew honey. According to these authors, honeydew honey exhibited a higher antioxidant potential than buckwheat honey, which is opposite to our previous results [17] and described in this paper’s findings and results obtained by other authors [31,34]. Furthermore, a very strong, positive correlation was observed between phenolic content and antioxidant activity (0.981, *p* < 0.05), which suggests that phenolics are mainly responsible for the antioxidant potential of honey, which was also previously confirmed by other authors [33,35].

### 2.2. GOX Activity

Glucose oxidase (GOX) is an enzyme sensitive to light and storage conditions and is activated after honey dilution, due to easy access to its substrate–glucose. GOX oxidizes glucose with the production of gluconic acid (the most representative acid in honey) and hydrogen peroxide [36]. Glucose oxidase activity determined in 20% honey solutions depends on the honey sample and ranged from 17 mU/mL (for goldenrod) to 140 mU/mL for one sample of honeydew honey (Table 2). Generally, the lowest GOX activity was observed for goldenrod honey, then for buckwheat honey. Manuka exhibited GOX activity similar to goldenrod honey. Huge differences in GOX activity among samples of the same honey variety (the highest observed for lime honey) was observed. Strelec et al. [37] demonstrated comparable GOX activity of honeydew and lime honey to our results. Bucekova et al. [38] observed that GOX activities in honeydew honey ranged from 21 to 50 mU/mL, which is lower than results presented in this paper for honeydew honey. However, the very low activity of GOX in manuka honey was similar to our results (<20 mU/mL). The authors suggest that methylglyoxal present in manuka honey may structurally modify the GOX enzyme. 

### 2.3. Hydrogen Peroxide Content

Data for hydrogen peroxide content are shown in Table 2. We can see that the level of hydrogen peroxide accumulated in diluted honey varies significantly and high differences can be observed between samples of the same honey variety. Similar results were observed by Bucekova et al. [30,38]. They observed that diluted blossom honey generates hydrogen peroxide at a concentration ranging from 32 to 3376 µM (100× difference between samples) and from 300 to 3400 µM for honeydew honey (more than 10× difference between honey samples). The maximum differences in hydrogen peroxide concentration presented in this work among the same honey types were around 200× those for lime honey. 

### 2.4. Antibacterial Activity

**Figure 1:**
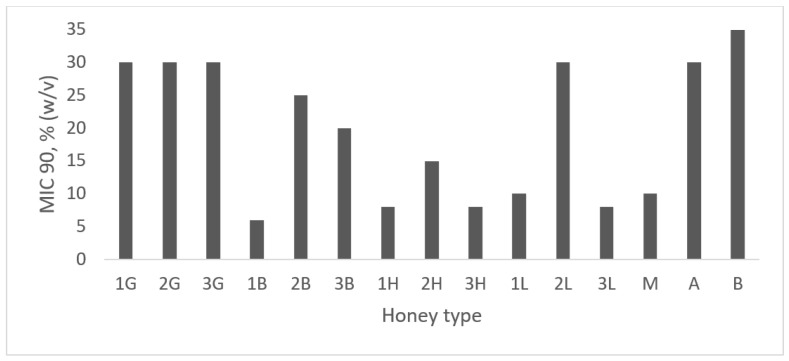
Antibacterial activity of honey samples, medical-grade manuka, and artificial honey against *Streptococcus mutans*. Activity was determined with a minimum inhibitory concentration (MIC). MIC was defined as the lowest concentration of honey solution (%) inhibiting bacterial growth at least 90% in comparison with positive control (100% growth of *S. mutans* on the medium without honey addition). 1–3G—goldenrod honey, 1–3B—buckwheat honey; 1–3H—honeydew honey; 1–3L—lime honey; M—manuka honey; A,B—artificial honey.

All analyzed honey samples inhibited *S. mutant’s* growth. The minimal inhibitory concentration (MIC) values are shown in Figure 1. The highest antibacterial potential was observed for honeydew honey with the lowest MIC value (8–15%). Generally, lime honey also strongly inhibits *S. mutans* growth, reaching MIC at the level of 8–10%, but with one exception (MIC value—30%). The MIC value of buckwheat honey samples varied from 6% (one honey) to 20–25% for other samples. Antibacterial properties of three samples of goldenrod honey were at the same level (MIC value—30%). Medical-grade manuka honey also strongly inhibited *S. mutans* growth (MIC = 10%), whereas Habluetzel et al. [23] determined weaker antibacterial potential of manuka honey at the level of 20% (*w/v*). Schmidlin et al. [22] demonstrated, by using the agar well diffusion method, the higher antibacterial potential of manuka honey in comparison with multifloral honey. As was expected, the weakest antibacterial potential was observed for artificial honey, where only the osmotic effect is considered. Similarly, Nassar et al. [20] compared artificial honey with natural honey (without mentioning the honey variety) and observed higher inhibition of *S. mutans* growth for natural honey than for the artificial one. In other publications, results vary widely; for example, Ahmadi-Motamayer et al. [39] showed that honey inhibited the growth of *S. mutans* at concentrations greater than 20%, while Ghabanchi et al. (2010) [40] showed inhibition of bacterial growth at 100% honey concentration. These results were obtained using the agar well diffusion method. Basson et al. [41] determined the antimicrobial potential of a few honey samples and also manuka honey, and in all cases reported that the MIC value was 25%. According to results proposed by Albaridi [18] regarding the division of antibacterial potential into three stages (depending on MIC % *w/v* values), all analyzed samples possessed strong (MIC between 1 and 12.5%) or moderate (MIC from 12.5% to 50%) antimicrobial potential against *S. mutans*. Antibacterial potential was significantly negatively correlated with total phenolic content (r = −0.567, *p* < 0.05), which means that the higher the phenolic content, the lower the MIC value. This high correlation indicates that polyphenols play a significant role in inhibiting the growth of *S. mutans*, which was also observed for other bacteria, like *Staphylococcus aureus* and *Pseudomonas aeruginosa* [30]. 

### 2.5. Antibacterial Activity of Honey after Enzymatic Treatment with Catalase and Proteinase K—The Role of Proteins and Hydrogen Peroxide in S. mutans Growth Inhibition

**Figure 2:**
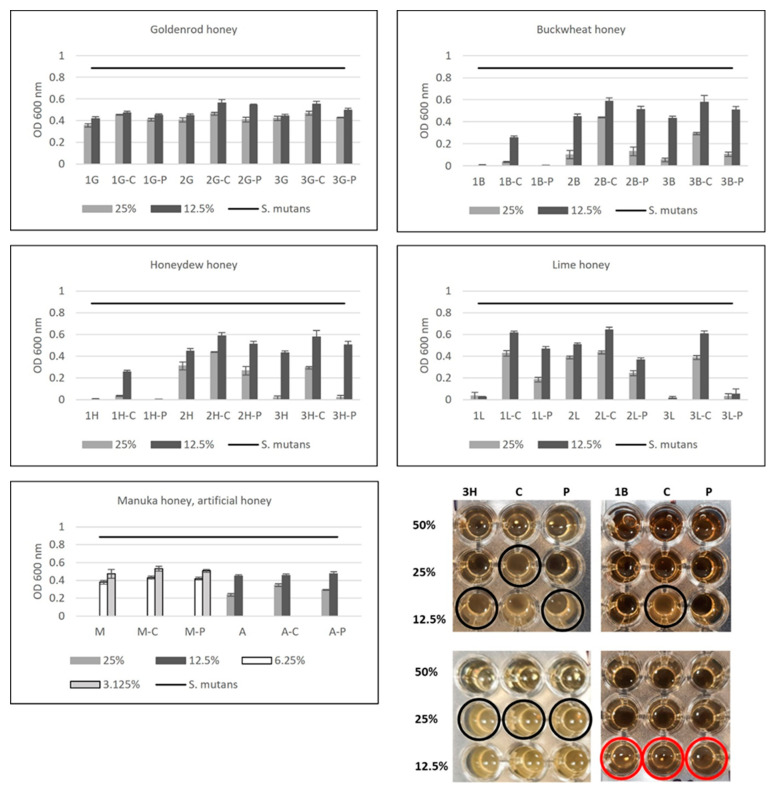
Influence of catalase and proteinase K treatment of honey on the *S. mutans* growth. The data present bacterial growth expressed as optical density (OD 600 nm) in the presence of honey (1–3G—goldenrod honey, 1–3B—buckwheat honey, 1–3H—honeydew honey, 1–3L—lime honey, M—manuka honey, A—artificial honey), without enzymes treatment and treated with catalase (C) and proteinase K (P). Control—*S. mutans* growth (without honey addition). The photo shows the growth of bacteria in contact with the selected enzyme treated and untreated honeys (3H—honeydew, 1B—buckwheat, 1G—goldenrod, M—manuka). The black circles indicate the lowest concentration of honey in which bacterial growth was observed. The red circles indicate no bacterial growth. The data are expressed as mean values with standard deviations (SDs).

In order to evaluate the influence of proteins and peptides, like glucose oxidase, on honey antibacterial potential against *Streptococcus mutans*, 50% honey solutions were treated with proteolytic enzyme proteinase K. It was proved by Bucekova et al. [30] that honey treatment with proteinase K caused complete digestion of the proteinous compounds present in honey. To define the role of hydrogen peroxide in antibacterial properties of honey against *S. mutans*, 50% of honey samples were treated with catalase (an enzyme disrupting hydrogen peroxide). Results for the antibacterial properties of proteinase K and catalase untreated and treated samples are shown in Figure 2. Comparing the antibacterial properties of 25% (*w/v*) solutions of honey, it can be observed that proteinase treatment did not influence the honey antibacterial potential in most analyzed samples. Only in the case of lime honey did proteinase K treatment decrease the antibacterial potential from 4 to 30% (depending on the sample). Similarly, Bucekova et al. [30,38] observed that proteinase K treatment of honeydew and blossom honey samples did not change their antibacterial potential against *Pseudomonas aeruginosa* and *Staphylococcus aureus*. Voidarou et al. [24] observed statistically significant reduced activity against oral pathogens after proteinase K treatment in 33% of analyzed honey samples. The obtained results suggest that glucose oxidase is not crucial for the antibacterial potential of honey, which was also confirmed by the lack of a statistically important correlation between these two factors (r = −0.171, *p* < 0.05).

On the other hand, catalase treatment significantly decreased the antibacterial potential of honey by around 35–50% for buckwheat, honeydew, and lime honey (in 25% honey solutions). A lower decrease in antibacterial potential was observed for goldenrod honey (15–25%). In Greek honey, catalase impact on its antimicrobial potential was variety-dependent (in citrus honey, antibacterial effect was significantly reduced, while remaining stable in oregano, sage, and *Satureja* spp. honey), but significant reduction in antibacterial properties was observed in all honey samples [24]. Therefore, a reduction in antibacterial activity after catalase treatment confirms that hydrogen peroxide plays an important role in antibacterial activity of honey against *S. mutans*, which was also supported by a strong, statistically important correlation between these parameters (r = −0.558, *p* < 0.05). Similar results were also observed by other researchers, who analyzed different honey samples against other bacteria [24,38,42]. Bucekova et al. [38] observed significant correlations between the antibacterial potential against *S. aureus* and *P. aeruginosa,* and the hydrogen peroxide content of blossom honeys. On the other hand, they did not observe any statistically important correlations between the antibacterial potential against *S. aureus* and *P. aeruginosa* and hydrogen peroxide content in honeydew honey [30]. Such differences may be explained by the fact that the kinetics of hydrogen peroxide production and degradation vary among honey samples. Samples with higher levels of H_2_O_2_ had later peaks in production than honey with lower levels of H_2_O_2_ [43]. Researchers mostly determine H_2_O_2_ content at one time point, after a different incubation time, which may have an important influence on obtained results. As was expected, catalase and proteinase K treatment of manuka honey did not change its antibacterial potential, which was also previously confirmed by Bucekova et al. [38]. 

In the photo presented in Figure 2 we can observe that in the case of honeydew sample 3H, catalase treatment caused bacterial growth at 25% concentration of honey, while at this concentration, in untreated and proteinase K treated honey, bacterial growth was not observed. Similarly, for the 1B (buckwheat) sample, bacterial growth was observed for the catalase-treated sample at 12.5% honey concentration, while in the untreated and proteinase-treated samples there was no bacterial growth. No differences in bacterial growth were observed for the 1G (goldenrod) sample and for manuka honey (no bacterial growth observed in enzyme treated and untreated samples). 

Moreover, no correlation was observed between GOX activity and hydrogen peroxide content (r = 0.005, *p* < 0.05). Similarly, Bucekova et al. [30,38] did not observe correlation between GOX activity and hydrogen peroxide content in blossom and honeydew honey. It is suggested that other substances present in honey participate in H_2_O_2_ production, which is crucial for honey antibacterial activities. Polyphenols can produce higher levels of hydrogen peroxide by its autoxidation [24,30,38]. This suggestion was also confirmed by an observed strong correlation between total phenolic content and the antibacterial potential of honey (r = −0.567; *p* < 0.05). Summarizing, the concentration of H_2_O_2_ is crucial to the antibacterial potential of honey against *S. mutans*, but hydrogen peroxide is generated not only due to GOX activity but also by phenolic compounds. 

### 2.6. Antibiofilm Activity

Bacteria developed some abilities, like biofilm formation, to help them survive in environments where antibiotics and other antimicrobial agents are present. Biofilm is a microbial community with the ability to adhere to solid surfaces and excrete extracellular polymeric substances (EPSs) which protect bacteria from stressful environmental factors and enable them to proliferate in the form of biofilm [5,44]. Cells in biofilm are 1000 times more resistant to antibiotics than cells in their planktonic state. Honey was found to reduce biofilm mass by killing bacterial cells entrapped in the biofilm matrix [45]. *S. mutans* is an important component of the biofilm on human teeth, which is directly associated with dental caries. Honey influence on *S. mutans* biofilm formation and biofilm degradation is presented in Table 3. It was shown that biofilm inhibition depends on honey concentration and on honey variety. The lowest inhibition of biofilm formulation can be observed for goldenrod honey. The antibiofilm properties of honeydew honey and buckwheat honey were comparable to manuka honey. As we expected, artificial honey also presented a very weak antibiofilm potential. The highest antibiofilm activities were observed for some samples of honeydew honey, buckwheat honey, and lime honey. Because of the fact that, even at the highest concentration of honey (50%), the maximum inhibition of biofilm formation for the “best” samples was around 70%, the results were expressed as MBIC 50 (minimal biofilm inhibitory concentration of honey, which inhibits at least 50% in comparison to a positive control (sample without honey addition)). The antibiofilm potential of honey samples was lower when previously established biofilm was used (Table 3). According to the last review article of Deglovic et al. (2022) [3], the antibiofilm activity of honey against periodontic bacteria in biofilm has not been intensively investigated, so it is very difficult to compare our results. However, this emphasizes the need to intensify research related to the possibilities of using honey in the treatment of dental caries. 

## 3. Materials and Methods

A total of 12 different types of honey were purchased directly from individual beekeepers located in southeastern Poland (Podkarpacie) in the beekeeping season of 2021. The botanical source of each honey type was performed by beekeepers, based on flora availability during the harvest season, the location of the apiary, and, in some, confirmed by pollen analysis of nectarous samples. Goldenrod (G) (*n* = 3), buckwheat (B) (*n* = 3), honeydew (H) (*n* = 3), and lime (L) honey were used. Manuka honey MGO 550+ was obtained from New Zealand Honey LTH and was used as a positive control. Artificial honey (purchased at a local store) was used as a negative control (sugar analog for the determination of antimicrobial activity to simulate the content of the main sugars in honey).

Honey samples were stored in glass containers at room temperature in the dark until analysis. The quality of the tested honeys was confirmed on the basis of physicochemical tests strictly defined in the EU Directive from 2014. The honeydew honey quality and authenticity were confirmed by measuring its electrical conductivity value (the minimum required is 0.8 mS/cm) and sensory judgement, because of the lack of internationally accepted quality criterion for honeydew honeys (Tomczyk et al., [27]).

### 3.1. Antioxidant Potential

#### 3.1.1. Total Polyphenol Content (TPC)

Total phenolic content was measured using the Folin–Ciocalteu colorimetric method according to Dżugan et al. [17] with minor modifications. Honey samples in the volume of 20 µL were mixed with 100 µL of 10% Folin–Ciocalteu reagent (Chempur, Piekary Slaskie, Poland) and 80 µL of 7.5% of sodium carbonate in 96-well microplate wells and incubated for 60 min. Absorbance was measured at 750 nm against blank (using a microplate reader). Gallic acid (Sigma Aldrich Co., St. Louis, MO, USA) was used for a calibration curve and results were expressed as mg gallic acid equivalent (GAE) per 100 g of the mass sample (mg GAE/100 g of honey).

#### 3.1.2. Antioxidant Potential Measured by FRAP 

Ferric reducing antioxidant power (FRAP) was used to determine the total antioxidant capacity (TAC) of the honey samples. The FRAP reagent contained 2.5 mL of 10 mM TPTZ (Sigma Aldrich Co, St. Louis, MO, USA) solution in 40 mM HCl (Chempur, Piekary Slaskie, Poland), 2.5 mL of 20 mM FeCl_3_ (Sigma Aldrich Co., USA), and 25 mL of a 0.3 M acetate buffer (pH 3.6) (Chempur, Piekary Slaskie, Poland). Aliquots of 0.02 mL of the honey solution (1 g/10 mL) were mixed with 0.18 mL of FRAP reagent in 96-well microplates, left for 10 min, and then the absorbance of the mixture was measured spectrophotometrically at 600 nm on a microplate reader against a blank. Trolox (Sigma Aldrich Co., USA) was used for the calibration curve and the results were expressed as mg of Trolox per 100 g of honey (mg Trolox/100 g of honey) [17]. 

### 3.2. Glucose Oxidase Activity Determination

Glucose oxidase (GOX) activity was determined with a Megazyme GOX assay kit (Megazyme International Ireland, Ltd., Bray, Ireland). The kit is based on two reactions–first, glucose is oxidized by glucose oxidase with the production of D-glucono-δ-lactone with a hydrogen peroxide release, which reacts with p-hydroxybenzoic acid and 4-aminoantipyrine, in the presence of peroxidase, to form a dye complex, whose absorbance is measured at 510 nm. For this purpose, 20% (*w/v*) of honey solutions were prepared and analysis was performed in a 96-well microplate, according to the manufacturer’s instructions. Results were expressed as mU/mL. 

### 3.3. Hydrogen Peroxide Determination

Hydrogen peroxide was determined according to Lehmann et al. [43]. Briefly, 5 g of a honey sample was diluted in 5 mL of distilled water prewarmed to 37 °C, then incubated for 20 min at 35 °C with shaking (180 rpm). Then, aliquots of 2.5 mL of honey samples were transferred to 28 mL McCartney bottles and further diluted to 25% (*w/v*) with water, a catalase solution (Sigma Aldrich Co., USA) (10 mM, phosphate buffer, pH 6.5 with catalase 2 mg/mL), and 10 mM of a phosphate buffer, pH 6.50 (a catalase blank solution). Samples, protected from light, were incubated at 35 °C in an orbital shaking incubator at 180 r.p.m. After 4 h of incubation, the hydrogen peroxide content was measured. For this purpose, a working reagent was prepared in 10 mM of a phosphate buffer pH 6.5 (18.96 mL) by adding 1 mL of O-dianisidine (1 mg/mL) (Sigma Aldrich Co., USA) and 40 µL of horseradish peroxidase type II (10 mg/mL) (Sigma Aldrich Co., USA). Then, 20 µL of each honey sample was added to a 96-well plate, mixed with 135 µL of working reagent, incubated at room temperature for 5 min, and then the reaction was stopped by adding 120 µL of 6 M sulfuric acid (Chempur, Piekary Slaskie, Poland). Absorbance was measured at 560 nm. For the calibration curve, different concentrations of hydrogen peroxide (Sigma Aldrich Co., USA) were prepared (0–2200 µg/mL). Results were expressed as µg of H_2_O_2_ in a 25% honey sample.

### 3.4. Antibacterial Properties of Honey—Minimum Inhibitory Concentration (MIC) 

The antibacterial activity of the different types of honey was tested against *S. mutans* PCM 2502 with the use of the microdilution method. Honey in a concentration of 50% (*w/v*) was prepared in a double-concentrated MHB medium (Mueller–Hinton Broth, Biomaxima, Poland), from which serial dilutions (ranging from 50 to 4%) were prepared in a MHB medium. Then, 200 µL of each honey sample was added to the wells of a microtiter plate and incubated with 20 µL of bacteria (from the overnight culture on a TSA medium (Trypticasein Soy Agar, Biomaxima, Poland), a bacterial concentration of 0.5 McFarland turbidity scale). Plates were incubated for 24 h at 37 °C without shaking. After incubation, bacterial growth was measured spectrophotometrically at 600 nm on a microplate reader. Appropriate controls were applied: positive control of culture growth (MHB medium without honey addition) and a negative control (a MHB medium without bacteria). The experiments were carried out in triplicate. Results were expressed as an MIC 90 value (minimal inhibitory concentration of honey which inhibits bacterial growth in ≥90%) in comparison to the corresponding positive control after 24 h of incubation (Grabek-Lejko et al. [15]). 

### 3.5. Antibacterial Activity of Honey after Enzymatic Treatment with Catalase and Proteinase K

Catalase, an enzyme that degrades hydrogen peroxide, was used in order to evaluate the contribution of hydrogen peroxide in the antibacterial activity of honey. Proteinase K (Sigma Aldrich Co., USA) was used to evaluate the contribution of proteins and peptides in the antibacterial potential of honey. For this purpose, a catalase stock solution was prepared by dissolving 30 mg of catalase from a bovine liver (Sigma Aldrich, Poland) in 10 mL of a 10 mM phosphate buffer (pH 7.4). To each 1.5 mL of 50% (*w/v*) solution of honey (dissolved in a Mueller–Hinton Broth medium (Biomaxima, Poland), 28 µL of the catalase stock solution was added. The stock solution of Proteinase K was prepared by diluting 10 mg proteinase K powder in 1 mL of distilled water, then adding it to honey samples to a final concentration of 100 µg/mL. Samples were incubated for 16 h at 37 °C in an incubator shaker with a rotation of 210 rpm. After incubation, the serial dilutions of honey were prepared in a Mueller–Hinton Broth medium, then 200 µL of diluted honey was added to each well in a 96-well microtiter plate. Then, 20 µL of a freshly prepared suspension of *S. mutans* (OD_600nm_ = 0.132, incubated on a TSA plate, 24 h, 37 °C) was added to each well. Then, samples were incubated at 37 °C without shaking. After 24 h of incubation, the optical density of the samples was measured at 600 nm by using a microplate reader. Controls without honey (positive growth control) and with catalase or proteinase K (catalase or proteinase K only control) were included to evaluate the effect of catalase/proteinase K alone on bacterial growth [30]. 

### 3.6. Biofilm Inhibition Activity

Antibiofilm activity was determined according to Haney et al. [46], Grabek-Lejko et al. [15], and Zayed et al. [47]. *S. mutans* was cultured overnight at 37 °C on a TSA medium. Then, bacterial suspension in TSB (Tryptic Soy Broth, Biomaxima, Poland) was prepared (0.5 McFarland scale). A series of diluted honey (50, 45, 40, 35, 30, 25, 20, 15, 10, 8, 6, and 4% *w/v*) in a TSB medium with 2% of sucrose were prepared and added, in the volume of 200 µL, to each well of a 96-well polystyrene microtiter plate, then 20 µL of the prepared bacterial culture was added. The negative control was a TSB+2% sucrose medium and the positive control (biofilm formation) was a bacterial culture in TSB+2% sucrose (without honey addition). Plates were incubated for 24 h at 37 °C without shaking. Then, the plates were processed as follows: the bacterial culture was decanted by inversion of the microtiter plate and the plates were washed three times with sterile saline (0.9%). Adherent cells were fixed with 200 µL of methanol and left at room temperature for 20 min. Then, the methanol was removed and plates were left to dry. Biofilms were dyed with 200 µL of a 0.2% crystal violet solution for 20 min. The redundant dye was removed by washing (3 times) with distilled water, then plates were dried and adherent cells with their formed biofilms were resolubilized by adding 200 µL of 33% glacial acetic acid and gently mixing. The absorbance of samples was measured at 600 nm using a microplate reader. The antibiofilm properties of honey were determined as MBIC 90 (minimal biofilm inhibitory concentration of honey that inhibits biofilm formation at least at 90% in comparison to control (amount of biofilm that was grown in the absence of honey, defined as 100% biofilm) [48]. Additionally, media sterility control samples were prepared (defined as 0% biofilm formation).

### 3.7. Biofilm Eradication Assay

To evaluate the effect of honey on preformed biofilms, first, 200 µL of bacterial suspension in TSB medium (prepared as previously described) was added to the wells of a 96-well microtiter plate. Plates were incubated for 24 h at 37 °C without shaking to allow biofilm attachment and growth. On the next day, medium and planktonic unbound cells were removed and the biofilm was washed three times with 200 µL of 0.9% NaCl. Then, appropriate diluted honey samples were added in the volume of 200 µL. For positive control, only a medium was added to the wells with biofilm. Plates were again incubated under static conditions (37 °C for 24 h). Then, the procedure of staining with crystal violet was repeated (as described above). The amount of biofilm inhibition was presented as MBIC 50 (minimal biofilm inhibitory honey concentration that eradicates biofilm at least at 50% in comparison to positive control (without honey addition; amount of biofilm growth defined as 100%). 

### 3.8. Statistical Analysis

All experiments were performed at least in triplicate. Mean values of the experiments were calculated and considered. Statistical analysis of the results was performed using Statistica v.13.3 (StatSoft, Inc., Tulsa, OK, USA). For all determinations, a one-way ANOVA was carried out using Duncan’s test at a significance level of *p* = 0.05. For the obtained data, mean values of the experiments were calculated and considered, and standard deviations were calculated. Pearson’s correlation coefficients (r) were calculated to show the significant differences between the selected data. Antibacterial correlation with other properties was calculated by comparing bacterial growth at 12.5% honey concentration. 

## 4. Conclusions

As a conclusion, we can say that these preliminary data suggest that Polish honey, especially honeydew honey and buckwheat honey, strongly inhibits *S. mutans* growth and inhibits biofilm formation. The antibacterial potential of the strongest honey samples was comparable to the higher levels of manuka activity. Moreover, the concentration of hydrogen peroxide and polyphenols plays an important function in the antibacterial potential of honey against *S. mutans*. These data are promising in the possible use of honey as a natural therapeutic agent in oral diseases. However, due to the small number of scientific papers describing the antibiofilm activity of honey against oral bacteria, additional research is needed to better understand the chemical components and mechanisms of the action of honey. 

## Figures and Tables

**Table 1:** Phenolic content and antioxidant properties of honey.

Honey Type	Honey Sample	TPC (mg GAE/100 g)	FRAP (µmol TE/100 g)
Goldenrod	1G	35.43 ± 1.52 ^c,d^	91.23 ± 1.41 ^a,b^
	2G	33.42 ± 0.60 ^b,c^	97.73 ± 5.66 ^a,b,c^
	3G	30.27 ± 0.71 ^a,b^	75.07 ± 3.61 ^a^
Buckwheat	1B	139.73 ± 3.23 ^j^	796.40 ± 43.24 ^k^
	2B	97.99 ± 5.00 ^i^	525.60 ± 7.63 ^i^
	3B	88.72 ± 4.43 ^h^	354.40 ± 35.36 ^g^
Honeydew	1H	64.25 ± 2.44 ^f^	343.60 ± 5.60 ^g^
	2H	69.27 ± 5.36 ^g^	343.60 ± 21.90 ^g^
	3H	98.72 ± 3.44 ^i^	584,13 ± 11.21 ^j^
Lime	1L	36.03 ± 2.93 ^c,d^	141.60 ± 4.92 ^d,e^
	2L	48.22 ± 2.38 ^e^	236.33 ± 9.97 ^f^
	3L	40.05 ± 2.00 ^d^	167.07 ± 16.15 ^e^
Manuka	M	84.75 ± 1.33 ^h^	403.60 ± 21.14 ^h^
Artificial	A	27.58 ± 0.42 ^a^	125.73 ± 0.83 ^c,d^
	B	28.22 ± 0.76 ^a^	122.00 ± 2.12 ^b,c,d^

Data are expressed as the mean values with standard deviation of the mean (SD). G—goldenrod honey, B—buckwheat honey, H—honeydew honey, L—lime honey, M—manuka honey, A,B—artificial honey. ^a–k^ Values with the same letters within the column are not significantly different (*p* < 0.05).

**Table 2:** Glucose oxidase (GOX) activity and hydrogen peroxide (H_2_O_2_) content in honey.

Honey Type	Honey Sample	H_2_O_2_ (umol/mL *)	GOX (mU/mL)
Goldenrod	1G	39.57 ± 0.49 ^c^	23.17 ± 0.35 ^c^
	2G	17.74 ± 1.12 ^b^	16.74 ± 0.01 ^b^
	3G	100.32 ± 17.33 ^d^	26.39 ± 0.58 ^c^
Buckwheat	1B	114.73 ± 4.52 ^e^	87.26 ± 2.92 ^g^
	2B	23.12 ± 0.99 ^b^	47.38 ± 0.94 ^e^
	3B	161.30 ± 1.05 ^f^	36.91 ± 1.93 ^d^
Honeydew	1H	161.29 ± 1.61 ^f^	70.73 ± 2.23 ^f^
	2H	164.52 ± 15.97 ^f^	84.53 ± 2.60 ^g^
	3H	16.67 ± 0.37 ^b^	139.14 ± 3.19 ^i^
Lime	1L	17.74 ± 0.32 ^b^	71.80 ± 4.44 ^f^
	2L	11.83 ± 0.93 ^b^	120.20 ± 4.53 ^h^
	3L	215.81 ± 7.38 ^g^	25.71 ± 0.59 ^c^
Manuka	M	16.88 ± 0.49 ^b^	17.22 ± 0.40 ^b^
Artificial	A	0.00 ± 0.00 ^a^	3.38 ± 0.40 ^a^
	B	0.00 ± 0.00 ^a^	3.45 ± 0.32 ^a^

Data are expressed as the mean values with standard deviations of the mean (SEMs). G—goldenrod honey, B—buckwheat honey, H—honeydew honey, L—lime honey, M—manuka honey, A,B—artificial honey; ^a–i^—Values with the same letters within the column are not significantly different (*p* < 0.05); *—results expressed in 25% honey concentration.

**Table 3:** Influence of honey on *S. mutans* biofilm formation and biofilm eradication. Results are expressed as MBIC 90 (minimal biofilm inhibitory concentration of honey, which inhibits biofilm formation at least 90%, in comparison to a bacterial sample without honey addition) and as MBIC 50 (minimal biofilm inhibitory concentration of honey, which inhibits eradication of biofilm at least 50% in comparison with bacterial sample without honey addition).

Honey Type	Honey Sample	Biofilm Formation MBIC 90	Biofilm Eradication MBIC 50
Goldenrod	1G	30	45
	2G	30	>50
	3G	30	50
Buckwheat	1B	15	10
	2B	30	40
	3B	30	45
Honeydew	1H	20	25
	2H	15	50
	3H	20	45
Lime	1L	25	25
	2L	30	>50
	3L	20	>50
Manuka	M	20	50
Artificial	A	30	>50
	B	35	>50

## Data Availability

Data are contained within the article.
